# Organism type of infection is associated with prognosis in sepsis: an analysis from the MIMIC-IV database

**DOI:** 10.1186/s12879-023-08387-6

**Published:** 2023-06-26

**Authors:** Qiuping Guo, Peng Qu, Wanfu Cui, Mingrong Liu, Huiling Zhu, Weixin Chen, Nan Sun, Shiyu Geng, Weihua Song, Xu Li, Anni Lou

**Affiliations:** 1 Department of Emergency Medicine, Southern Medical University Nanfang Hospital, Guangzhou, 510515 China; 2 Department of Gastroenterology, Guangdong Provincial Key Laboratory of Gastroenterology, Nanfang Hospital, Southern Medical University, Guangzhou, 510515 China; 3 Key Laboratory of Emergency and Trauma, Ministry of Education, College of Emergency and Trauma, Hainan Medical University, Haikou, 571199 China

**Keywords:** Sepsis, Gram-positive bacteria, Gram-positive bacteria, Fungi, Prognosis, MIMIC-IV

## Abstract

**Background:**

Sepsis has a high mortality rate, which is expensive to treat, and is a major drain on healthcare resources; it seriously impacts the quality of human life. The clinical features of positive or non-positive blood cultures have been reported, but the clinical features of sepsis with different microbial infections and how they contribute to clinical outcomes have not been adequately described.

**Methods:**

We extracted clinical data of septic patients with a single pathogen from the online Medical Information Mart for Intensive Care(MIMIC)-IV database. Based on microbial cultures, patients were classified into Gram-negative, Gram-positive, and fungal groups. Then, we analyzed the clinical characteristics of sepsis patients with Gram-negative, Gram-positive, and fungal infections. The primary outcome was 28-day mortality. The secondary outcomes were in-hospital mortality, the length of hospital stay, the length of ICU stay, and the ventilation duration. In addition, Kaplan–Meier analysis was used for the 28-day cumulative survival rate of patients with sepsis. Finally, we performed further univariate and multivariate regression analyses for 28-day mortality and created a nomogram for predicting 28-day mortality.

**Results:**

The analysis showed that bloodstream infections showed a statistically significant difference in survival between Gram-positive and fungal organisms; drug resistance only reached statistical significance for Gram-positive bacteria. Through univariate and multivariate analysis, it was found that both the Gram-negative bacteria and fungi were independent risk factors for the short-term prognosis of sepsis patients. The multivariate regression model showed good discrimination, with a C-index of 0.788. We developed and validated a nomogram for the individualized prediction of 28-day mortality in patients with sepsis. Application of the nomogram still gave good calibration.

**Conclusions:**

Organism type of infection is associated with mortality of sepsis, and early identification of the microbiological type of a patient with sepsis will provide an understanding of the patient's condition and guide treatment.

## Introduction

Sepsis is a life-threatening organ dysfunction caused by a dysregulated host response to infection and is the main cause of death occurring in ICU patients [1]. Sepsis is an enormous threat to human life and health, especially in developing countries where medical resources are lacking [2]. It is estimated that the total number of sepsis cases worldwide was 48.9 million in 2017, of which about 11 million were sepsis-related deaths, accounting for 19.7% of the total global deaths [3]. In recent years, with the publication of sepsis guidelines, timely and effective antibiotics, and other comprehensive management treatments continue to improve, and the mortality rate of sepsis has decreased but remains at a high level [4–6]. High cost of sepsis treatment and a huge drain on medical resources. At present, the diagnosis and treatment of sepsis remain a serious concern and challenge in hospitals.

Infection is one of the causes of sepsis, and bacteria and fungi are the most common pathogenic microorganisms of infection [7]. Different types of infection may have different outcomes for the disease. Several studies have identified the ability of positive microbiological cultures to influence prognosis and laboratory tests in patients with sepsis [8, 9]. However, studies on the impact of different pathogen types on disease outcomes are relatively scarce and the findings are somewhat controversial; A meta-analysis including 510 studies found that mortality rates were significantly higher in Gram-negative bacteremia than in Gram-positive bacteremia patients, suggesting a significant impact of microbial type on sepsis prognosis [10]. However, Zahar et al. concluded that there was no correlation between different pathogenic microorganisms and sepsis prognosis [11]. Early identification of the organism type of infection can help clinicians understand the patient's condition, enabling them to give adequate attention and adjust treatment plans in time.

Therefore, we used the Medical Information Mart for Intensive Care (MIMIC)-IV, a large publicly available database for critical care in the United States, from which we extracted relevant clinical information to compare the clinical characteristics and 28-day all-cause mortality of septic patients with Gram-positive, Gram-negative, and fungal infections and to further clarify the impact of different pathogens on the prognosis of septic patients.

## Materials and methods

### Study design

This is a retrospective case–control study of data from the online critical care medicine database—MIMIC-IV, which is a relational database containing real hospital stays for patients admitted to a tertiary academic medical center in Boston, MA, USA. MIMIC-IV provided critical care data for over 40,000 patients admitted to intensive care units at the Beth Israel Deaconess Medical Center (BIDMC), which contains complete information about each patient during their hospitalization, including laboratory measurements, medications administered, vital signs documented, and so on [12]. The data for this study came from the MIMIC public database and has obtained ethical approval from the institutional review boards of the Massachusetts Institute of Technology and Beth Israel Deaconess Medical Center (BIDMC), so patient consent or ethical approval was not required for this study.

An individual who has finished the Collaborative Institutional Training Initiative(CITI) examination (Certification number 42805075 for author Guo) can access the database.

### Inclusion and exclusion criteria

Patients meeting sepsis 3.0 diagnostic criteria in the database were screened for analysis. The inclusion criteria were as follows: (1) patients who met the sepsis 3.0 criteria or had a sepsis diagnosis in the discharge diagnosis in the ICD code; (2) age ≧ 18 years; (3) length of ICU stay ≧ 24 h; (4) microbiological testing was performed within 48 h before and after the ICU admission. Exclusion criteria: (1) for patients with multiple ICU admissions, only data from the first ICU admission were included; (2) patients with culture-negative microorganisms or canceled tests; (3) patients with multiple (≧ 2)infection types.

### Data extraction

In this study, patient parameters were extracted, including baseline characteristics such as age, gender, race, body mass index (BMI), first 24 h vital signs such as temperature, heart rate, respiratory rate, mean blood pressure, SpO 2, disease severity scores such as sequential organ failure (SOFA) score, simplified acute physiology II (SAPS II) score, comorbidities such as Charlson comorbidity score, myocardial infarction(MI), chronic congestive heart failure(CHF), chronic pulmonary disease(CPD), renal disease, tumor, etc., laboratory tests such as white blood cells(WBC), platelets, hemoglobin, blood lactate, blood creatinine, etc., and life support treatments, such as first 24 h renal replacement therapy(RRT), first 24 h mechanical ventilation, and the use of vasopressors. Temperature, heart rate, respiratory rate, and mean blood pressure were taken as the average values on the first day of ICU admission, and SpO_2_ was taken as the worst value. Laboratory test indexes were the worst values on the first day of ICU admission. Vasopressors included norepinephrine, phenylephrine, epinephrine, vasopressin, dopamine, and dobutamine. The code for data extraction is available from GitHub (https://github.com/MIT-LCP/mimic-iv).

### Outcomes

The primary outcome in this analysis was 28-day mortality with different microbial types of infection. The secondary outcomes were in-hospital mortality, the length of hospital stay, the length of ICU stay, and ventilation duration.

## Statistical analysis

Continuous variables are expressed as mean ± SD or median (IQR), as appropriate. Categorical variables were expressed as numbers (percentages). Student's t-test, Mann–Whitney U test, ANOVA, and Kruskal–Wallis test followed by post hoc Bonferroni test for multiple comparisons was used for continuous variables. X^2^ test or 2-tailed Fisher exact test followed by post hoc Bonferroni test for multiple comparisons was used for categorical variables. Survival curves were calculated using the Kaplan–Meier method and the log-rank test.

We implemented the univariate logistic regression analysis to investigate the relationship between different variables and 28-day mortality, and the potential confounders that were considered clinically relevant or showed univariate relationships with the 28-day mortality at a significant level (*p* < 0.1) were entered into the multivariate logistic regression analysis as covariates. We calculated the Receiver Operating Characteristic(AUC) Curve to assess the sensitivity and specificity of the model for predicting 28-day mortality. Then, we developed and validated a nomogram based on the results of multivariate logistic regression analysis for the individualized prediction of 28-day mortality in patients with sepsis.

We used the software R 4.1.3, GraphPad prism 8.0, and spss26.0 for data analysis. Statistically significant was defined as a *P* value < 0.05.

## Results

### Population characteristics

**Fig. 1:**
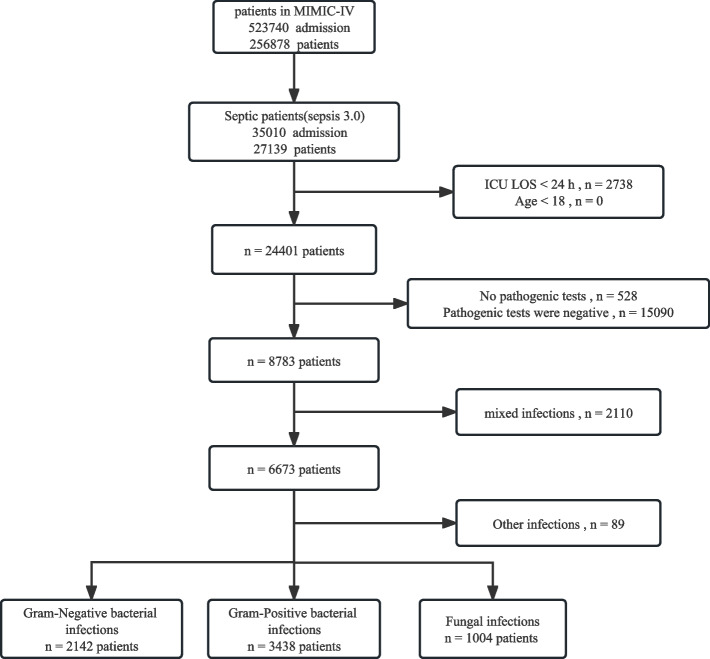
Flowchart of study cohort selection

A total of 27,139 patients met the diagnosis of sepsis 3.0 in the MIMIC-IV database. After a rigorous screening process, 6584 patients were included in this study (Fig. 1). There were 2142 cases in the Gram-negative bacteria (GNB) group, 3438 cases in the Gram-positive bacteria (GPB) group, and 1004 cases in the fungal group. Baseline data and clinical characteristics of the patients are shown in  Table 1.

**Table 1:** Baseline demographic and clinical characteristics by organism type in patients with sepsis

	Total (*n* = 6584)	Gram-negative bacterium (*n* = 2142)	Gram-positive bacterium (*n* = 3438)	Fungus (*n* = 1004)	*p* value
Age(years)	67.1 ± 16.7	70.2 ± 16.2	65.7 ± 16.9	65.5 ± 16.3	< 0.001^a,b^
Male (%)	3508 (53.3)	1013 (47.3)	2015 (58.6)	480 (47.8)	< 0.001^a,c^
White (%)	4439 (67.4)	1475 (68.9)	2311 (67.2)	653 (65.0)	0.096
Weight (kg)	81.9 ± 28.8	79.6 ± 31.3	83.5 ± 28.1	81.4 ± 25.1	< 0.001^a,c^
**Vital signs**					
Temperature (℃)	37.0 ± 0.6	37.0 ± 0.6	37.0 ± 0.6	36.9 ± 0.7	0.157
HR (bpm)	88.8 ± 16.9	87.9 ± 16.9	88.5 ± 16.7	91.4 ± 17.2	< 0.001^b,c^
RR(bpm)	20.3 ± 4.2	20.4 ± 4.0	20.1 ± 4.2	21.0 ± 4.4	< 0.001^a,b,c^
MBP (mmHg)	76.2 ± 10.3	75.7 ± 9.8	76.7 ± 10.6	75.7 ± 10.2	0.001^a,c^
SpO2 (%)	92(90–95)	92 (90–94)	92 (90–95)	92 (88–94)	< 0.001^a,c^
**Severity of illness**					
SOFA	7.1 ± 3.9	6.9 ± 3.9	6.7 ± 3.7	8.6 ± 4.2	< 0.001^a,b,c^
SAPSII	41.6 ± 14.6	42.4 ± 14.6	40.0 ± 14.3	45.4 ± 14.7	< 0.001^a,b,c^
Septic shock (%)	880 (13.4)	314 (14.7)	406 (11.8)	160 (15.9)	< 0.001^a,c^
Charlson comorbidity score	6.2 ± 3.0	6.3 ± 2.9	6.0 ± 3.0	6.4 ± 3.1	< 0.001^a,c^
**Comorbidities, n (%)**					
MI	1183 (18)	381 (17.8)	637 (18.5)	165 (16.4)	0.304
CHF	2149 (32.6)	670 (31.3)	1136 (33)	343 (34.2)	0.21
CPD	1876 (28.5)	575 (26.8)	961 (28)	340 (33.9)	< 0.001^b,c^
Renal disease	1624 (24.7)	515 (24)	872 (25.4)	237 (23.6)	0.376
Tumor	1072 (16.3)	382 (17.8)	489 (14.2)	201 (20)	< 0.001^a,c^
Severe liver disease	529 (8)	143 (6.7)	268 (7.8)	118 (11.8)	< 0.001^b,c^
Diabetes	2163 (32.9)	660 (30.8)	1157 (33.7)	346 (34.5)	0.045
AIDS	50 (0.8)	13 (0.6)	24 (0.7)	13 (1.3)	0.098
**Laboratory tests**					
Lactate (mmol/L)	3.1 ± 2.8	3.3 ± 2.9	3.0 ± 2.6	3.0 ± 2.9	0.022^a^
HGB (g/dL)	9.8 ± 2.1	9.9 ± 2.1	9.9 ± 2.1	9.5 ± 2.1	< 0.001^b,c^
PLT (× 10^9^/L)	187.4 ± 110.5	179.4 ± 102.1	187.4 ± 108.1	204.7 ± 132.1	< 0.001^a,b,c^
WBC (× 10^9^/L)	16.1 ± 10.5	16.1 ± 12.1	15.9 ± 9.7	16.7 ± 9.4	0.097
BUN (mg/dL)	34.2 ± 26.3	34.1 ± 25.9	33.5 ± 26.2	36.9 ± 27.4	0.002^b,c^
Creatinine (mg/dL)	1.8 ± 1.8	1.7 ± 1.5	1.9 ± 2.0	1.9 ± 1.8	0.003^a,b^
Lymphocytes (× 10^6^/L)	82.5 ± 219.1	70.6 ± 97.5	90.7 ± 285.2	79.3 ± 118.4	0.001^a^
Neutrophils(× 10^6^/L)	723.2 ± 847.2	716.7 ± 913.4	713.7 ± 793.7	768.4 ± 879.9	0.312
NLR	16.0 ± 34.9	17.5 ± 30.9	14.4 ± 21.5	18.6 ± 65.6	0.043^a,c^
**Need of life support, n (%)**					
Vasopressor	3428 (52.1)	1081 (50.5)	1718 (50)	629 (62.6)	< 0.001^b,c^
MV of first 24 h	3207 (48.7)	899 (42)	1643 (47.8)	665 (66.2)	< 0.001^b,c^
RRT of first 24 h	395 (6)	95 (4.4)	199 (5.8)	101 (10.1)	< 0.001^b,c^
**Primary source of infection, n (%)**					
Bloodstream	1426 (21.7)	405 (18.9)	976 (28.4)	45 (4.5)	< 0.001^a,b,c^
Respiratory tract	1931 (29.3)	582 (27.2)	718 (20.9)	631 (62.8)	< 0.001^a,b,c^
Genitourinary	2231 (33.9)	1120 (52.3)	797 (23.2)	314 (31.3)	< 0.001^a,b,c^
Skin and soft tissue	287(4.4)	48(2.2)	227(6.6)	12(1.2)	< 0.001^a,c^
Gastrointestinal	223 (3.4)	33 (1.5)	185 (5.4)	5 (0.5)	< 0.001^a,b,c^
Abdomen	68 (1)	30 (1.4)	24 (0.7)	14 (1.4)	< 0.001^a^
Others/unspecified	1015(15.4)	22(1.0)	980(28.5)	13(1.3)	-

Data are reported as the mean ± SD or median (interquartile range)

*BMI* Body mass index, *HR* Heart rate, *RR* Respiratory rate, *MBP* Mean blood pressure, *SOFA* Sequential organ failure assessment, *SAPSII* Simplified acute physiology score II, *MI* Myocardial infarction, *CHF* Congestive heart failure, *CPD* Chronic pulmonary disease, *AIDS* Acquired immune deficiency syndrome, *HGB* Hemoglobin, *PLT* Platelet, *WBC *White blood cell, *NLR* Neutrophil to lymphocyte ratio, *RRT* Renal replacement therapy, *MV* Mechanical ventilation

The *p*-value represents the result of comparing Gram-negative bacteria, Gram-positive bacteria and fungi

^a^Gram-negative bacteria vs Gram-positive bacteria (*p* < 0.05)

^b^Gram-negative bacteria vs Fungus (*p* < 0.05

^c^Gram-positive bacteria vs Fungus (*p* < 0.05

BMI (body mass index) index was excluded from this study as height data were missing in 2629 patients. Of the 6584 patients included in this study, the mean(SD) age was 67.1 ± 16.7 years, 3508 (53.3%) patients were male and 4439 (67.4%) patients were white persons. The SOFA score and SAPS II score were 7.1 ± 3.9 and 41.6 ± 14.6 on average, respectively. The fungal group had worse SOFA and SAPS II scores than the GNB and GPB groups, with pairwise comparisons showing statistically significant differences. The incidence of septic shock was 14.7%, 11.8%, and 15.9% in GNB, GPB, and fungal groups, respectively, with the gram-positive bacteria group having the lowest incidence(*p* < 0.05). The common sources of infection were the genitourinary tract in 2231 (33.9%), the respiratory tract in 1931 (29.3%), and the bloodstream in 1426 (21.7%). Compared to GNB and GPB groups, a larger percentage of the use of vasopressors(50.4% vs. 50% vs. 62.6%, *p* < 0.05), first 24 h mechanical ventilation(42% vs. 47.8% vs. 66.2%, *p* < 0.05), and first 24 h RRT(4.4% vs. 5.8% vs. 10.1%, *p* < 0.05) in the fungal group. There were no significances in diabetes, AIDS, MI, CHF, renal disease, neutrophils, and neutrophil to lymphocyte ratio(NLR) on ICU admission among the three groups.

### Distribution of bacterial species and infection site

**Fig. 2:**
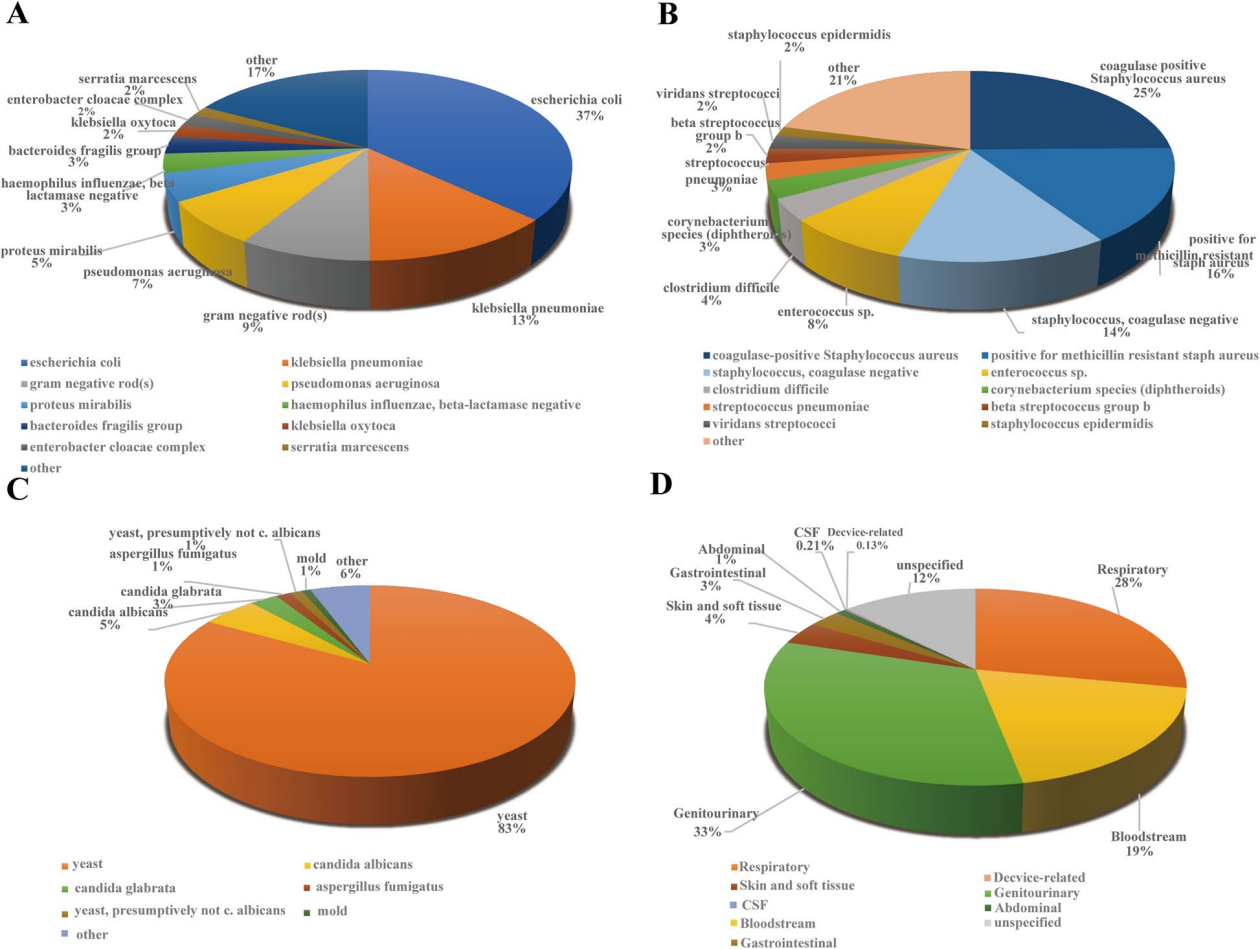
Primary bacterial species and infection site Gram-negative bacterium (**A**), Gram-positive bacterium (**B**), Fungus (**C**), infection site (**D**)

This study depicts the common pathogen species and sites of GNB, GPB, and fungi infection in patients with sepsis. The most frequently isolated GNB in sepsis is *Escherichia coli* (37%), followed by *Klebsiella pneumoniae* (13%), and *Pseudomonas aeruginosa* (7%) (Fig. 2A). *Staphylococcus aureus*(25%), followed by *methicillin-resistant staphylococcus aureus*(16%) and *coagulase-negative staphylococcus*(14%), etc. are the most common pathogens among the GPB (Fig. 2B). Among the fungi, yeast (83%) makes up the most prevalent pathogen, next by *Candida albicans* (5%), *Candida smoothies* (3%), and *Aspergillus fumigatus* (1%)(Fig. 2C). The genitourinary tract(33%), trailed by the respiratory tract(28%) and bloodstream infection (19%), was the most typical location of the infection (Fig. 2D).

### Clinical outcomes of different organism types of infection

Different organism types of infection can result in different clinical outcomes. The 28-day mortality, in-hospital mortality, length of hospital stay, length of ICU stay, and ventilation duration for all patients included were 19.1%, 19.5%, 9.8 days, 3.6 days, and 46.1 ± 90.5 (SD) hours, respectively(Table 2). In comparison to the GNB and GPB groups, patients in the fungal group had greater 28-day mortality (31.8% vs. 15.4% vs. 17.7%), in-hospital mortality (32.6% vs. 15.8% vs. 18.0%), and ventilation duration (77.2 vs. 40.0 vs. 40.9 h, *p* < 0.05). The GPB group had higher rates of 28-day mortality and in-hospital mortality than the GNB group (*p* 0.05). However, the hospital stay of GPB was shorter than the GNB groups (9.5 vs. 13.2 days, (*p* < 0.05)(Table 2).

**Table 2:** Primary and secondary outcome

	**Total (*n* = 6584)**	**Gram-negative bacterium (*n* = 2142)**	**Gram-positive bacterium (*n* = 3438)**	**Fungus (*n* = 1004)**	***p*-value**
**28-day mortality (%, n/N)**	19.1 (1257/6584)	15.4 (328/2142)	17.7(610/3438)	31.8 (319/1004)	*p* < 0.001^a,b,c^
**In-hospital mortality (%, n/N)**	19.5 (1284/6584)	15.8 (337/2142)	18.0 (620/3438)	32.6 (327/1004)	*p* < 0.001^a,b,c^
**Length of hospital stay (d)**	9.8 (5.9, 16.9)	13.2(5.6, 15.6)	9.5(5.9, 16.6)	13(7.4, 21.5)	*p* < 0.001^a,b,c^
**Length of ICU stay (d)**	3.6 (2.0, 7.0)	5.6(1.9, 6.6)	3.4(2.0, 6.4)	5.3(3.0, 9.8)	*p* < 0.001^b,c^
**Ventilation duration**	46.1 ± 90.5	40.0 ± 89.3	40.9 ± 82.7	77.2 ± 110.4	*p* < 0.001^b,c^

^a^Gram-negative bacterium vs Gram**-**positive bacterium (*p* < 0.05

^b^Gram-negative bacterium vs Fungus (*p* < 0.05)

^c^Gram-positive bacterium vs Fungus (*p* < 0.05)

### Subgroup analyses

**Fig. 3:**
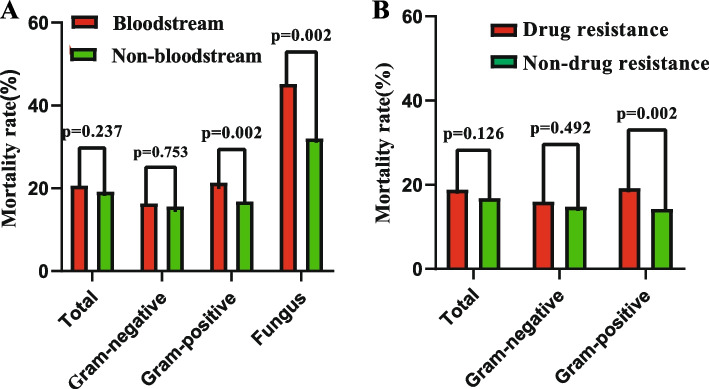
Subgroup analyses

The study was further analyzed in subgroups for drug-resistant bacteria and bloodstream infections. There was no significant difference in 28-day mortality in the GNB (P = 0.753) and overall (*P* = 0.237) groups among bloodstream and non-bloodstream infections. However, for the GPB and fungal groups, the 28-day mortality rate was higher in the bloodstream infection group than in the non-bloodstream infection group, both *p*-values were equal to 0.002 (Fig. 3A. Meanwhile, the difference in 28-day mortality between drug-resistant and non-drug-resistant patients was not statistically significant in the overall (*P* = 0.412)and GNB groups (*P* = 0.496), but drug-resistant patients in the GPB group had an increased 28-day mortality rate than non-drug-resistant patients (*P* = 0.002) (Fig. 3B).

### Overall survival and effect of organism type of infection on survival

**Fig. 4:**
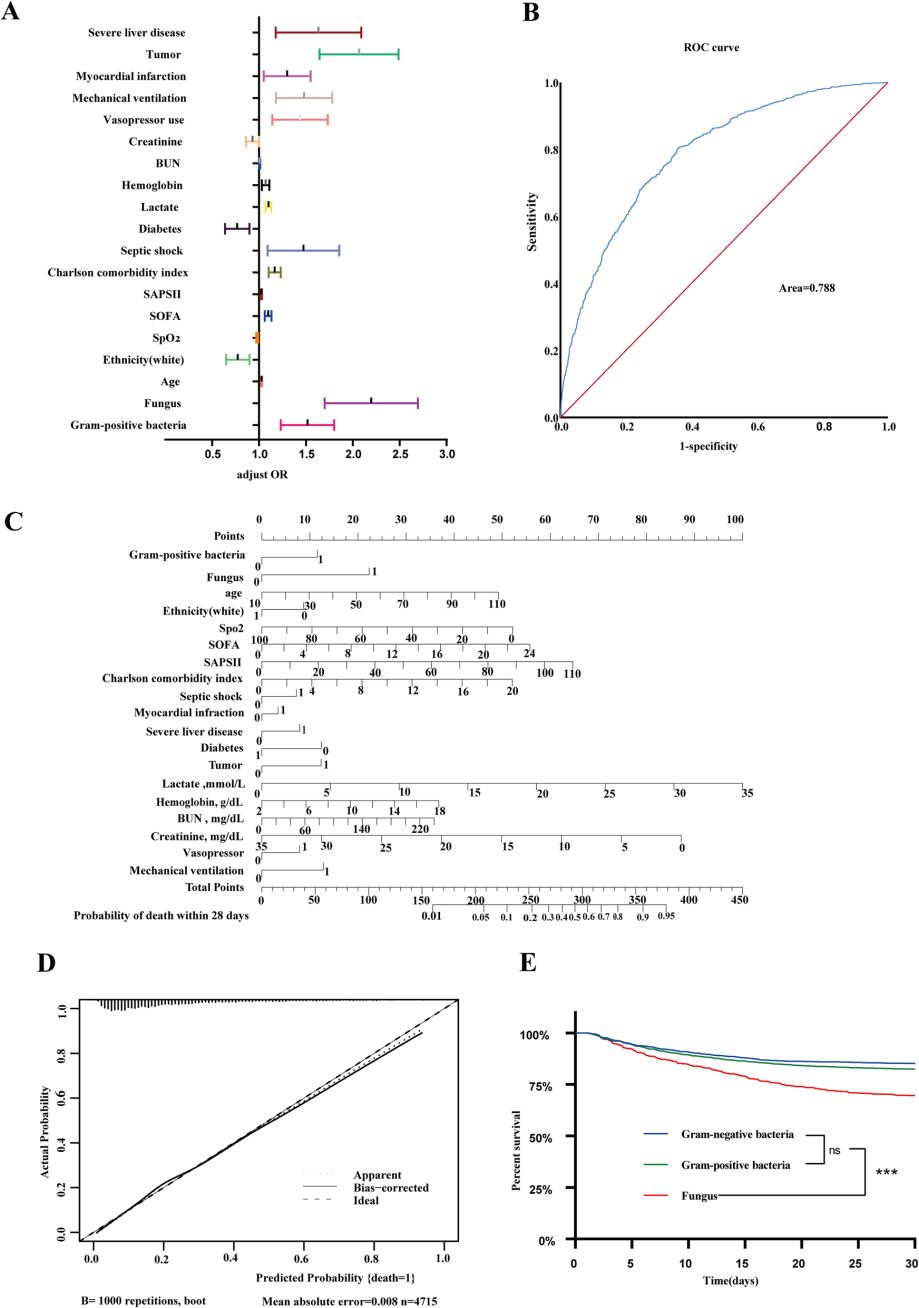
Forest diagram of multivariate regression analyses of variables for 28-day Mortality (**A**), The performance of the model was tested internally, and receiver operating characteristic (ROC) curve analysis is used to discriminate model performance analysis (**B**). A Nomogram for predicting 28-day mortality (**C**), Calibration curves of the nomogram in the internal validation cohort (**D**), and Kaplan–Meier analysis for 28-day survival. KM survival curve analysis of the 28-day survival rates of the three groups (**C**). HR1:Gram-negative bacterium vs Fungus, HR2:Gram-positive bacteria vs Fungus (**E**)

By using univariate and multivariate logistic regression models, our research further examined whether the microbial types were independent risk factors for 28-day mortality events. In a univariate logistic regression model, using the Gram-negative group as a reference, the probability of the endpoint event was 1.22(95% CI 1.05–1.42, *P* = 0.009) and 2.48(95% CI 2.07–2.97, *P* < 0.001) times higher in the GPB and fungal groups, respectively (Table 3) (Fig. 4A). Similarly, after adjusting for covariates, GPB and fungi remained significant independent risk factors for 28-day mortality on multivariable analysis, 1.50(95% CI 1.24–1.81), 2.16 (95% CI 1.72–2.7) respectively (Table 3). The multivariate logistic model showed good discrimination, with a C-index of 0.788 (Fig. 4B). We then developed and validated a nomogram for the individualized prediction of 28-day mortality in septic patients (Fig. 4C). Application of the nomogram still gave good discrimination (C-index, 0.778) and good calibration (Fig. 4 D.

**Table 3:** Univariate and multivariate regression analyses of variables for 28-day mortality

Parameter	Univariate model		Multivariate model	
	OR(95%CI)	*p* value	OR(95%CI)	*p* value
Gram-negative bacterium	Reference	-	Reference	-
Gram-positive bacterium	1.22(1.05–1.42)	0.009	**1.50(1.24–1.81)**	**< 0.001**
Fungus	2.48(2.07–2.97)	< 0.001	**2.16(1.72–2.71)**	**< 0.001**
Infect site(bloodstream)	1.04(0.90–1.21)	0.592	-	
Drug resistance	0.92(0.77–1.09)	0.337	-	
Age	1.02(1.02–1.02)	< 0.001	**1.03(1.02–1.03)**	**< 0.001**
Gender (male)	1.01(0.89–1.14)	0.934	-	
Ethnicity(white)	0.78(0.68–0.88)	< 0.001	**0.77(0.65–0.90)**	**0.001**
MBP	0.98(0.98–0.99)	< 0.001	1.00(0.99–1.01)	1.000
SpO_2_	0.96(0.95–0.97)	< 0.001	0.98(0.97–0.99)	**< 0.001**
SOFA	1.22(1.20–1.24)	< 0.001	**1.10(1.06–1.13)**	**< 0.001**
SAPS II	1.06(1.06–1.07)	< 0.001	**1.02(1.02–1.03)**	**< 0.001**
Charlson comorbidity index	1.16(1.13–1.18)	< 0.001	**1.17(1.10–1.23)**	**< 0.001**
Septic shock	1.48(1.25–1.75)	< 0.001	**1.44(1.11–1.87)**	**0.006**
Myocardial infarction	1.50(1.29–1.75)	< 0.001	**1.28(1.06–1.56)**	**0.012**
Congestive heart failure	1.18(1.04–1.35)	0.012	0.85(0.71–1.02)	0.079
Chronic pulmonary disease	1.07(0.93–1.22)	0.351	-	
Renal disease	1.15(1.00–1.32)	0.05	1.02(0.83–1.25)	0.846
Severe liver disease	1.90(1.56–2.33)	< 0.001	**1.59(1.20–2.11)**	**< 0.001**
Diabetes	0.89(0.78–1.02)	0.084	**0.76(0.64–0.90)**	**0.02**
Tumor	1.92(1.65–2.23)	< 0.001	**2.04(1.66–2.50)**	**< 0.001**
Aids	0.72(0.32–1.61)	0.424	-	
Lactate,mmol/L	1.20(1.17–1.23)	< 0.001	**1.10(1.07–1.13)**	**< 0.001**
Hemoglobin, g/dL	0.96(0.93–0.99)	0.01	1.07(1.03–1.11)	**0.01**
Platelets, × 10^9^/L	1.00(1.00–1.00)	< 0.001	1.00(1.00–1.00)	0.071
WBC, × 10^9^/L	1.01(1.01–1.02)	< 0.001	0.99(0.99–1.00)	0.368
BUN, mg/dL	1.01(1.01–1.01)	< 0.001	1.01(1.00–1.01)	**0.005**
Creatinine, mg/dL	1.09(1.06–1.12)	< 0.001	0.93(0.86–1.00)	**0.036**
Lymphocytes, × 10^9^/L	1.00(1.00–1.00)	0.267	-	
Neutrophils, × 10^9^/L	1.00(1.00–1.00)	0.242	-	
NLR	1.00(1.00–1.00)	0.114	-	
Vasopressor use	3.00(2.62–3.45)	< 0.001	**1.42(1.15–1.74)**	**0.001**
Renal replacement therapy	2.15(1.72–2.69)	< 0.001	1.06(0.76–1.49)	0.730
Mechanical ventilation	2.79(2.43–3.21)	< 0.001	**1.46(1.19–1.79)**	**< 0.001**

Kaplan–Meier survival curve demonstrating the organism type of infection as a risk factor for mortality. There is a significant survival disadvantage for patients with sepsis due to fungal infections within 28 days of hospitalization compared to Gram-positive or Gram-negative organisms(log-rank test: *P* < 0.001) (Fig. 4E). The HRs for the occurrence of 28-day mortality for fungi versus GNB and GPB were 1.818 (95% CI, 1.574–2.100; *P* < 0.001) and 1.715 (95% CI, 1.494–1.968; *P* < 0.001), respectively (Fig. 4E).

## Discussion

In this retrospective cohort study, we found that the type of infection plays an important role in the pathogenesis of sepsis and is one of the most important independent risk factors for endpoint events in patients with sepsis. The risk of short-term mortality was higher for bloodstream infections caused by fungi and GPB; Multidrug-resistant GPB have a higher mortality rate than non-drug-resistant patients. Finally, we also developed and validated clinical prediction nomograms for the 28-day mortality in patients with sepsis, and it performed well with good calibration.

Our current understanding of the pathogenesis of sepsis is based on the lipopolysaccharide(lps)-induced cell model, which may not be suitable for GPB and fungi because of the variability in the clinical manifestations of different microbial infections [13]. Moreover, GPB and GNB lead to different immune mechanisms. Earlier research has shown that *Staphylococcus aureus* (47.3%), *Enterococcus spp* (10.8%), and *Candida spp* (10.1%) were the most common infections [14], which is consistent with our findings. GNB are more likely to cause severe sepsis than GPB, with higher SOFA, SAPS II, and Charlson comorbidity scores, but their prognosis of them is the opposite. A study pointed out that the APACHE II score was also significantly higher in the Gram-negative patients’ group than in the Gram-positive patients’ group [15].

Bloodstream infections and drug resistance are important factors that influence the management of sepsis. A subgroup analysis of our study showed that bloodstream infections are associated with poor disease prognosis mainly in GPB and fungi, and drug resistance mainly in GPB. Not all cases of sepsis are caused by bloodstream infections. In fact, bloodstream infections account for only 25–30% of sepsis, 21.7% in this study [16]. Bacteremia is associated with both short as well as long-term patient prognosis [17]. With the widespread use of antibiotics, resistance is a major challenge in the treatment of sepsis [18], and current treatment difficulties may lie mainly in the resistance of GPB, such as *methicillin-resistant Staphylococcus aureus* [19, 20]. The latest guidelines of the Surviving sepsis campaign (SCC) have highlighted that the early use of antimicrobial drugs is recommended for adult patients with highly suspected sepsis [21] and timely antifungal therapy is also important [22]. Therefore, the early identification of microorganisms by multiple blood cultures will facilitate the overall assessment of the disease and quick adequate antimicrobial therapy [23].

The mechanism for poor outcomes with Gram-positive and fungi organisms is not completely clear. In general, it may be explained by the structure and toxicology of the different pathogenic microorganisms and the response of the host [24]. It has been shown that GNB produce a more intense inflammatory response than GPB [15]. As we all know, fungi are more frequently seen in immunocompromised patients with tumors, severe liver disease, diabetes, and AIDS, which may explain the poorer prognosis of fungal than bacterial infections. In our study, age, AIDS, tumor, severe liver disease, NEU and other indicators reflecting immune status were included in univariate and multivariate regression. The results showed that gram-positive bacterial infection and fungal infection were still independent risk factors for the prognosis of sepsis. It is undeniable that there are other potential influencing factors, and further multicenter, prospective studies are needed to verify this result. GNB produce endotoxin mainly by lysis of the bacterium and GPB secrete exotoxin [25] to cause harm to the host. In addition, fungi are eukaryotic organisms with a nuclear envelope, it is hard to develop drugs to kill or control them without being harmful to humans [26].

With the introduction of sepsis 3.0, the previous sofa score may not adequately reflect the overall condition of the disease, and further improvement is needed. Over the years, several studies have explored the new risk factors associated with the prognosis of sepsis [27, 28]. Two observational studies found serum albumin to be a risk factor for death in patients with sepsis [29, 30]. Jang DH et al. reported that serum phosphorus levels were an independent risk factor for prognosis in 3034 patients with sepsis. High serum phosphorus levels suggested a poor prognosis [31]. One study comparing 1-year survival in patients with different platelet levels found platelet abnormalities to be a molecular marker of prognosis in sepsis [32]. To the best of our knowledge, we are the first to consider the type of microbial infection as one of the screening variables and have found it to be a meaningful indicator.

This study has several limitations as follows. Firstly, the study is a single-center retrospective clinical analysis and there may be some variation in the management of sepsis in different countries and regions, which needs to be validated using external data. Secondly, this database was established largely based on the surgical ICU which constituted of patients after surgical operation. So the findings may not be extrapolated to all kinds of septic patients. Thirdly, patients with positive etiological tests might not accurately represent the cause of their infection; colonization bacteria and opportunistic pathogens may have interfered. Additionally, there are instances of multiple infections or other pathogens such as viruses and parasites in the clinical environment, and our assessment of infections caused by a single pathogen has some limits. At last, despite the large sample size of this study, some prospective studies are required to further validate the accuracy of the results.

## Conclusions

In conclusion, our retrospective analysis shows a 1.5-fold and 2.1-fold increased adjusted risk for 28-day mortality in septic patients with GPB and fungi respectively, compared to a control group with GNB infections. We elucidated that the type of microorganism was independently associated with increased mortality. A nomogram can be conveniently used for individualized prognosis prediction and provide treatment advice for patients with sepsis. The exact mechanisms and pathophysiological differences between pathogenic microbial species are not well understood and this needs to be further investigated.

## Data Availability

The [DATA TYPE] data used to support the findings of this study are available from the corresponding author upon request.
